# Ventricular Volumetric Changes After Transcatheter Tricuspid Valve Replacement in a Heart Transplant Recipient

**DOI:** 10.1016/j.jaccas.2026.108819

**Published:** 2026-07-08

**Authors:** Denizhan Ozdemir, Mandeep Singh, Farhan A. Bajwa, Jean-Pierre Iskandar, Elizabeth C. Melito, Katherine Kaproth-Joslin, Kazuhiro Hisamoto, Leway Chen, Frederick S. Ling

**Affiliations:** University of Rochester Medical Center, Rochester, New York, USA

**Keywords:** computed tomography,, transcatheter tricuspid valve replacement, tricuspid regurgitation,, valvular disease

## Abstract

**Background:**

Severe tricuspid regurgitation is a common complication after orthotopic heart transplantation (OHT), but transplant recipients are underrepresented in transcatheter trials. Early ventricular volume and ejection fraction changes after transcatheter tricuspid valve replacement (TTVR) in this population remain unclear.

**Case Summary:**

A 62-year-old man with nonischemic cardiomyopathy, and subsequent OHT developed NYHA functional class III symptoms from severe tricuspid regurgitation. Transcatheter edge-to-edge repair was deemed unfeasible because of imaging limitations. Computed tomography demonstrated annular dilation, and TTVR with a 44-mm EVOQUE valve was performed. At 30 days, the valve was well functioning, without significant residual regurgitation or hypoattenuated leaflet thickening. Repeat computed tomography showed reduced biventricular end-diastolic volumes, with increased left ventricular and effective right ventricular ejection fractions.

**Discussion:**

This case supports feasibility and early physiologic directional ventricular changes after EVOQUE implantation in an OHT patient.

**Take-Home Message:**

TTVR is safe in OHT recipients, with early favorable changes in biventricular volumes and function.

## Visual Summary

**Figure:**
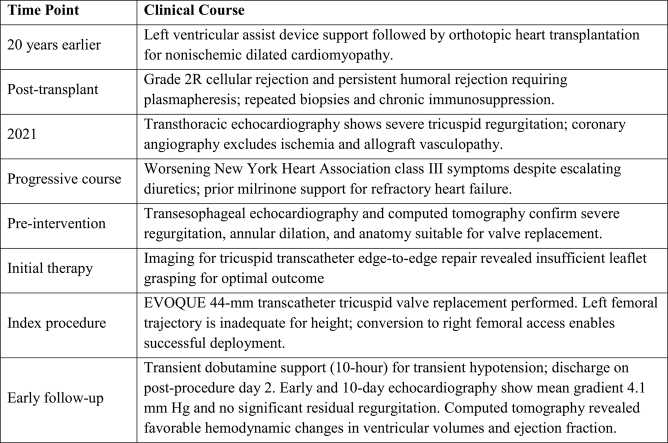

## History of Presentation

A 62-year-old man presented with progressive exertional dyspnea, fatigue, and reduced exercise tolerance consistent with NYHA functional class III heart failure. Examination and echocardiography were consistent with severe tricuspid regurgitation (TR) and right-sided volume overload.

**Take-Home Messages**

• EVOQUE valve replacement was feasible and safe in a heart transplant recipient.
• Early ventricular volumetric changes and improvements in right and left ventricular function demonstrate favorable hemodynamic effects after TTVR in a heart transplant recipient.

## Past Medical History

His history was notable for left ventricular assist device support followed by orthotopic heart transplantation 20 years earlier for nonischemic dilated cardiomyopathy. The posttransplant course had been complicated by International Society for Heart and Lung Transplantation grade 2R cellular and persistent humoral rejection requiring plasmapheresis. He was managed with chronic immunosuppression and escalating diuretic therapy; milrinone had previously been required for refractory heart failure. Coronary angiography showed no allograft vasculopathy or ischemic explanation for his symptoms.

## Differential Diagnosis

The differential diagnosis for worsening heart failure symptoms after transplantation included severe TR, right ventricular dysfunction, rejection-related graft dysfunction, constrictive physiology, pulmonary hypertension, and ischemia from allograft vasculopathy.

## Investigations

Transthoracic echocardiography in 2021 showed severe TR. Transesophageal echocardiography demonstrated severe regurgitation between the septal and anterior leaflets with annular dilation and central malcoaptation (vena contracta 0.7 cm, Video 1). The right ventricle was mildly dilated with mildly reduced longitudinal systolic function (right ventricular end-diastolic diameter 49 mm; tricuspid annular plane systolic excursion 17 mm). The etiology of TR is likely a combination of repeated endomyocardial biopsies and ventricular functional remodeling. Because surgery was declined, transcatheter therapy was pursued. Transcatheter edge-to-edge repair was attempted first, but aborted because intraprocedural imaging was inadequate for leaflet grasping. Preprocedural cardiac computed tomography (CT) then showed marked annular dilation (area 14.1 cm^2^; septal-lateral diameter 47 mm), supporting consideration of transcatheter tricuspid valve replacement (TTVR).

## Management

**Figure 1:**
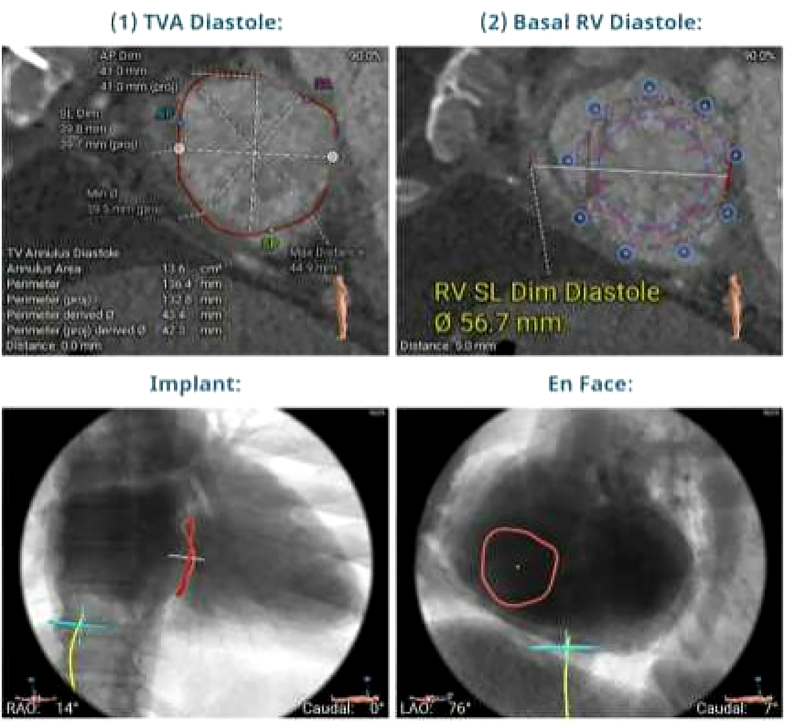
Computed Tomography Analysis for Transcatheter Tricuspid Valve Replacement RV = right ventricle; TVA = tricuspid valve atresia.

**Figure 2:**
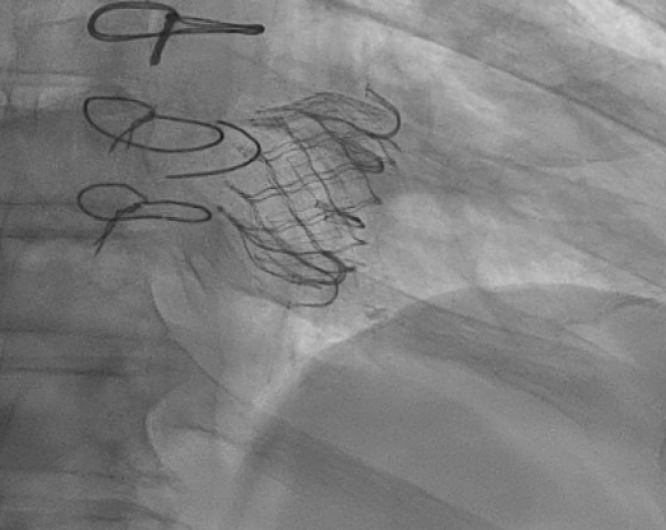
Fluoroscopic Deployment of the EVOQUE Valve

The procedure was performed under general anesthesia with transesophageal and intracardiac echocardiographic guidance. Bilateral femoral venous access was obtained. A Safari XS wire was positioned in the right ventricular apex. A 44-mm EVOQUE valve was selected based on CT analysis (Figure 1). Initial deployment attempts from the left femoral venous approach were unsuccessful because the delivery catheter trajectory was suboptimal and adequate depth across the annulus could not be achieved. The access strategy was therefore changed to the right femoral vein. With improved alignment, the prosthesis was advanced across the tricuspid annulus and deployed under 3-dimensional transesophageal echocardiographic guidance with satisfactory leaflet capture and depth. Final imaging showed excellent prosthetic position without paravalvular leak or residual significant TR (Figure 2, Video 2). Hemostasis was achieved with predeployed Perclose ProGlide closure devices. The patient remained in sinus rhythm with his preexisting right bundle branch block and no new conduction abnormality.

## Outcome and Follow-Up

**Figure 3:**
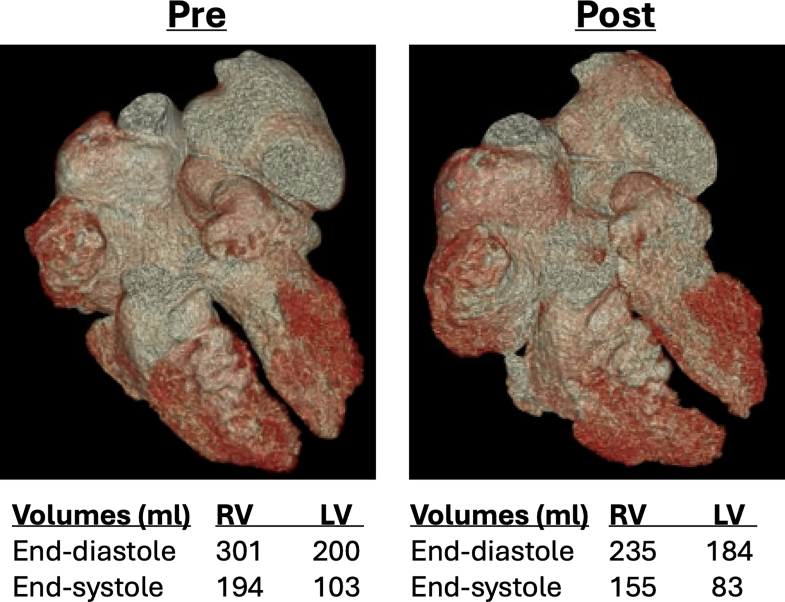
Ventricular Volumetric Changes Before and After Transcatheter Tricuspid Valve Replacement LV = left ventricle; RV = right ventricle.

After valve implantation, the patient developed mild hypotension requiring low-dose dobutamine, consistent with transient right ventricular afterload mismatch after abrupt elimination of severe TR. Transthoracic echocardiography showed no pericardial effusion. Dobutamine was discontinued within 10 hours, and he was discharged on postprocedure day 2 with ambulatory rhythm monitoring and no evidence of a new conduction abnormality. After multidisciplinary discussion with the heart transplant team, the decision was made to continue aspirin 81 mg daily for allograft vasculopathy prophylaxis. Apixaban was added to the patient's home regimen to reduce the risk of leaflet thrombosis. In the absence of randomized evidence, we plan to maintain aspirin 81 mg daily and direct oral anticoagulation for at least 6 months. The patient underwent mobile cardiac outpatient telemetry, which revealed no evidence of conduction disturbance; therefore, pacemaker implantation was not required. Early postoperative and 10-day follow-up echocardiography showed a well-functioning prosthesis with a mean transvalvular gradient of 4.1 mm Hg and no significant residual TR. Thirty-day imaging and functional follow-up showed similar findings. Given the unique nature of this case, CT evaluation was obtained at 30 days to assess for hypoattenuated leaflet thickening, which showed no evidence of leaflet thrombosis. It also revealed that the 3-dimensional right ventricular end-diastolic volume had decreased from 301 mL preprocedurally to 235 mL (Figure 3). Right ventricular end-systolic volume decreased from 194 mL to 155 mL (Figure 3). There was also a mild decrease in left ventricular end-diastolic volume, from 200 mL to 184 mL (Figure 3). Left ventricular end-systolic volume similarly decreased mildly, from 103 mL to 83 mL (Figure 3). Left ventricular ejection fraction increased mildly from 48% to 54%. Right ventricular ejection fraction (RVEF) changed from 35% preprocedurally to 33% postprocedurally. However, the preprocedural RVEF likely overestimated effective forward right ventricular systolic function because it included both forward stroke volume into the right ventricular outflow tract and regurgitant volume across the tricuspid valve. Following TTVR, the measured RVEF more accurately reflected effective RVEF, representing predominantly antegrade forward flow without significant regurgitant volume. Therefore, the apparent mild reduction in RVEF likely reflects unmasking of true forward right ventricular systolic function rather than procedural deterioration.

## Discussion

TR is increasingly recognized after orthotopic heart transplantation and may arise early from primary graft dysfunction or later from annular remodeling, repeated endomyocardial biopsy trauma, rejection, and progressive right ventricular dysfunction.^1^ In transplant recipients, redo surgery is often unattractive because of cumulative operative risk, whereas transcatheter edge-to-edge repair may be considered if anatomy demonstrates favorable findings.^2^^,^^3^

This case is notable for 3 reasons. First, it demonstrates the feasibility of TTVR with EVOQUE in a heart transplant recipient after suboptimal anatomy for transcatheter tricuspid edge-to-edge repair. Transplant recipients are commonly excluded from major transcatheter tricuspid trials; however, evidence is rapidly emerging that TTVR can be safely employed in suitable patients.^4^, ^5^, ^6^

Second, the need to anticipate transient hemodynamic deterioration after abrupt elimination of severe TR is well known.^7^ Temporary inotropic support may be required because the right ventricle is suddenly exposed to increased effective afterload.^7^ However, after the initial temporary adjustment to new volume-pressure mismatch, 30-day repeat CT revealed favorable cardiac remodeling in native hearts.^8^

Current expert guidance supports individualized transcatheter therapy for severe symptomatic TR in anatomically suitable patients who are poor surgical candidates.^9^ In that framework, valve replacement may be preferable when leaflet grasping is not feasible or annular and coaptation dimensions are prohibitive for edge-to-edge repair.^9^ Our case expands the limited literature supporting EVOQUE as a potential option in selected transplant recipients, but longer follow-up is needed to define durability, reverse remodeling, valve hemodynamics, and rhythm outcomes in this high-risk population.

**Equipment List:** Key Equipment Used During Transcatheter Tricuspid Valve Replacement

Device/Equipment	Purpose
18-F right femoral venous sheath	Primary venous access
12-F left femoral venous sheath	Contralateral venous access for intracardiac echocardiography
24-F Gore DrySeal sheath	Large-bore venous access after procedural site conversion
Safari XS wire	Right ventricular apical rail for device delivery
10-F intracardiac echocardiography catheter	Intraprocedural right-sided imaging
44-mm EVOQUE valve and delivery system	Transcatheter tricuspid valve replacement
Perclose ProGlide closure devices	Venous hemostasis at access sites

## Conclusions

TTVR with EVOQUE was feasible in this heart transplant recipient with severe symptomatic TR. At 30-day follow-up, TTVR was associated with early directional changes in ventricular volumes and ejection fractions. Given the short follow-up duration and the load-dependent nature of ventricular volumes and ejection fraction, these findings should be interpreted as early physiologic changes rather than definitive evidence of favorable ventricular remodeling.

## Funding Support and Author Disclosures

The authors have reported that they have no relationships relevant to the contents of this paper to disclose.

## Appendix

Video 1 Severe Tricuspid Regurgitation on Baseline Transesophageal Echocardiography

Video 2 Elimination of Tricuspid Regurgitation After TTVR TTVR, Transcatheter Tricuspid Valve Replacement.
